# Acknowledgment to the Reviewers of *Epidemiologia* in 2022

**DOI:** 10.3390/epidemiologia4010004

**Published:** 2023-01-16

**Authors:** Epidemiologia Editorial Office

**Affiliations:** MDPI AG, St. Alban-Anlage 66, 4052 Basel, Switzerland

High-quality academic publishing is built on rigorous peer review. *Epidemiologia* was able to uphold its high standards for published papers due to the outstanding efforts of our reviewers. Thanks to the efforts of our reviewers in 2022, the median time to first decision was 37.5 days and the median time to publication was 60.5 days. Regardless of whether the articles they examined were ultimately published, the editors would like to express their appreciation and thank the following reviewers for the time and dedication that they have shown *Epidemiologia*:
Ágnes, CsivincsikMadadizadeh, FarzanAli, AshaqMancon, AlessandroAl-sayyed, HibaMańczuk, MartaArguni, EggiManne, KartikAtalan, AbdulkadirMansor, RozaihanBaccolini, ValentinaMcCartney, GerryBailey, EmilyMendy, Vincent LutherBanerjee, AnkonaMilenković, BranislavaBieńkowski, CarloMonteiro, LuísButt, Muhammad HammadMúñez, ElenaBuzhilova, Alexandra P.Muñoz-García, Claudia I.Canora Lebrato, JesusNišavić, Jakov J.Cardoso, RodrigoNovozhilov, Artem S.Cerda, ArcadioOtrisal, PavelChodick, GabrielPanthee, BimalaDa Mota, Jurema CorrêaPertea, MihaelaDartnall, ElizabethPrasad, VibhuDelgado-Gallegos, Juan LuisPrestileo, TullioDianatinasab, MostafaRamalho, Alanderson AlvesDileepan, MythiliRizzo, EmanueleEl Hidan, Moulay AbdelmonaimRocha, Ian Christopher N.Elfaky, MahmoudRodic, AndjelaFal, AndrzejRodríguez-Hurtado, DianaFilho, Arnaldo Jorge MartinsSà, FilipaFranco, ChristianSanz Muñoz, IvanGanusov, VitalySchultz, DavidGarcia Cabrera, EmilioSilva, CândidaGentile, Stéphanie G.Silva, Gustavo Sousa E.Gupta, RituSilverii, Antonio GiovanniGutkowska, OlgaSong, KunGyawali, BishalSzczepaniak, KlaudiuszIoanăș, CorinaTakita, MorihitoIqhrammullah, MuhammadThevkar Nagesh, PrashanthIslam, Md. TaohidulTok, Peter Seah KengJagielska, AnnaTroup, LucyJain, Hemant KUllah, QudratJin, YingUpadhyay, Sushil K.Jurčev Savičević, AnamarijaVilinová, KatarínaKapczuk, PatrycjaVillani, Emanuele RoccoKarmaoui, AhmedWaap, HelgaKundnani, Nilima RajpalWang, BoLăzureanu, Voichița ElenaYakoob, Mohammad YawarLi, XinleiYin, XinLópez-García, XoséZhang, TianouLundin, RebeccaZincir, Handan

